# Application of One-Step Nucleic Acid Amplification (OSNA) in different cancer entities and usefulness in prostate cancer: a systematic review

**DOI:** 10.1186/s12885-022-09355-0

**Published:** 2022-04-02

**Authors:** Mercè Cuadras, Jacques Planas, Ana Celma, Lucas Regis, Inés M. de Torres, M. Eugenia Semidey, Enrique Trilla, Juan Morote

**Affiliations:** 1 Urology Department, Vall d’Hebron University Hospital, Barcelona, Spain; 2 Pathology Department, Vall d’Hebron University Hospital, Barcelona, Spain; 3 Surgery Department, Universitat Autònoma de Barcelona, Barcelona, Spain

**Keywords:** One-step nucleic acid amplification, OSNA, Cytokeratin 19, CK19, Prostate cancer, Lymph node metastases

## Abstract

**Background:**

Lymph node (LN) status is a key prognostic factor in the decision-making process of different cancer entities, including prostate cancer (PCa). Sectioning and haematoxylin and eosin (H&E) staining technique remain the gold standard for the evaluation of LN metastases despite some limitations, especially low sensitivity in detecting an accurate tumour burden within the LN, as well as a subjective and time-consuming result. One-step nucleic acid amplification (OSNA) quantifies mRNA copies of cytokeratin 19 (CK19) in a fast, objective, automated, and reproducible way, raising a general interest to explore its utility for lymphatic metastasis identification in different malignancies.

**Methods:**

To present the latest evidence related to the detection of LN metastases in several tumours by using OSNA compared with the conventional H&E method, a systematic review of articles published since March 2021 was conducted using PubMed, Cochrane Library, and Web of Science databases. References from primary papers and review articles were checked to obtain further potential studies. Our procedure for evaluating records identified during the literature search followed the Preferred Reporting Items for Systematic Reviews and Meta-analyses criteria. With the aim to design and justify future clinical routine use of OSNA in PCa, novel PCa evidence has been included in this review for the first time.

**Results:**

Twenty five studies were included. LN from six different groups of tumours: breast, gastrointestinal, gynecological, lung, head and neck and prostate cancers has been assessed. OSNA was compared with post-operative formalin-fixed paraffin-embedded tissue sections with H&E staining as the reference standard. Contingency tables were created, and concordance rate, sensitivity, specificity and predictive values were reported. Seventeen studies analysed the discordant cases using different techniques.

**Conclusion:**

OSNA method has a high diagnostic accuracy for the detection of LN metastases in several CK19 expressing tumours. Available evidence might encourage future investigations about its usage in PCa patients to improve LN staging and prognosis.

## Background

Prostate cancer (PCa) is the second most incident neoplasm and the fifth cancer specific cause of male mortality worldwide [1]. Upon diagnosis, PCa is classified into major risk categories based on TNM clinical stage, biopsy Gleason score, and serum prostate specific antigen (PSA) levels. High-risk patients associate more biochemical recurrence, metastatic progression, and PCa related death [2].

Pelvic lymph nodes (LN) represent the most common site of metastases in PCa patients considered for surgical treatment. According to the series reviewed, the risk of LN invasion at radical prostatectomy ranges between 3 and 24%, and could be even higher in high-risk PCa patients [3].

Conventional imaging techniques, such as computed tomography and magnetic resonance imaging, have low sensitivity for the detection of LN metastases [4]. The introduction of positron emission tomography with different radiotracers such as 11C-Choline and especially 68Ga-PSMA has increased the sensitivity to detect LN metastases. The 68Ga-PSMA has demonstrated > 90% specificity with sensitivity rates of 33–99% depending on serum PSA [5]. As ≤5 mm metastases are mostly missed by these techniques [6], extended pelvic lymph node dissection (ePLND) remains the most accurate staging procedure despite the fact that up to 20% of patients will present some kind of complication after its performance [7].

Due to the limited sensitivity of imaging techniques in the detection of small metastases, different nomograms based on preoperative characteristics have been described in order to define which PCa patient will truly benefit from an ePLND [8, 9].

Lymphadenectomy extent and histological nodal evaluation have an impact on the staging and consequent prognosis of the disease. The gold-standard procedure consists of a macroscopic identification of the LN, followed by its sectioning into 3–4 mm slices, and then analysis through haematoxylin and eosin (H&E) staining of at least one slice per LN [10]. Main limitations of this approach are metastatic tissue allocation and interobserver bias, as well as being costly and time-consuming.

New methods, such as serial section analysis (slices with a thickness of 1–2 mm), immunohistochemistry (IHC), and molecular tissue analysis using Reverse Transcription-Polymerase Chain Reaction (RT-PCR) for PSA have demonstrated a higher sensitivity to identifying low tumour burden in the nodes [11]. High costs, the time required for the analysis, and some limitations to standardization have hindered their routine application, though they remain relevant in clinical research.

In 2008, an innovative biomolecular technique called One-Step Nucleic Acid Amplification (OSNA) was introduced in Europe to assess LN metastases. OSNA is an automated system based on reverse transcription loop-mediated isothermal amplification method, able to quantify copies of cytokeratin 19 (CK19) mRNA. CK19 is a marker expressed by several solid tumours of epithelial origin, but not by healthy lymphatic tissue [12]. OSNA allows a quick and accurate analysis of the tumour burden of entire LN tissue in an objective, automated, and reproducible way [13–15]. It has been proven useful in different cancer entities, such as breast, colorectal, gastric, endometrial, cervical, lung, and head and neck cancer, achieving a high sensitivity and specificity in the detection of LN involvement, as well as a high concordance compared to comprehensive histopathological examination, in some cases even comparable to ultra-staging [16].

OSNA was first applied in the intraoperative analysis of sentinel lymph node (SLN) in breast cancer, introducing an objective evaluation of the nodal tissue, as well as reducing the required time and effort by the laboratory personnel. More than 10 years ago, Tsujimoto et al. [15] demonstrated the correlation between OSNA and conventional histopathological analysis of the SLN in breast cancer and defined the cut-off values for the distinction between macrometastases, micrometastases, and unaffected tissue. Since then, more than 200 studies have been published and the application range of OSNA was extended to other cancer entities [17].

The available scientific and clinical evidence, together with the mentioned characteristics, has introduced OSNA in current national and European clinical guidelines as an alternative technique for the determination of lymphatic involvement in breast cancer through SLN analysis [18]. Moreover, data available from studies in colorectal cancer demonstrated that OSNA is a valid technique for the detection of lymphatic involvement also in this cancer entity [19]. Hence, OSNA is now included in the recommendations for the determination of biomarkers in colorectal carcinoma [20].

Interestingly, the quantitative outcome of the OSNA assay was identified as useful tool to predict, during surgery, non-SLN involvement in breast and gynecological cancer, thus supporting tailoring of surgical procedure [21]. In breast and colorectal cancer, OSNA was shown to provide also prognostic information [22].

Main advantages and disadvantages of OSNA assay are summarized in Table 1.

**Table 1:** Advantages and disadvantages of OSNA

Advantages	Disadvantages
Fast, objective, automated, and reproducible technique	Not valid for non-CK19 expressing tumours
Intraoperative analysis	Trained pathologist needed (thorough dissection)
Analysis of the whole LN	Potential contamination of the sample
Quantitative analysis: • Cut-off points for macro and micrometastases • TTL: potential predictive and prognostic factor	Not applicable in case of coexisting neoplasms with the same LN drainage
Ability to a more accurate identification of micrometastases	No tissue left to re-analysis (except RNA-based molecular tests)

Regarding urological tumours, based on previous studies that demonstrated the expression of CK19 in PCa tissue, Winter et al. showed that OSNA method can detect CK19 mRNA in 100% of primary PCa tumours regardless of Gleason score and even more effectively than CK19 IHC expression, suggesting the valid application of this technique in LN evaluation [23]. In a very recent study, Engels et al. [24] demonstrated that OSNA can identify nodal metastases at an equivalent or, in cases of micrometastases, better rate than enhanced histological examination in PCa patients, confirming its promising use in intraoperative decision-making in personalized LN surgery.

To set up future clinical use of OSNA in PCa, the aim of this review is to analyse the available evidence of this technique in different tumours and propose short-term course of actions to transfer the validated concepts and successes from the other malignancies to PCa.

## Methods

### Search strategy

To retrieve all relevant papers published before the end of March 2021, three databases including PubMed, Cochrane Library, and Web of Science were searched by two independent reviewers combining the following Medical Subject Headings: one-step nucleic acid amplification, OSNA, lymph nodes, lymph node metastases, cytokeratin 19, CK19. References from primary papers and review articles were checked to obtain further potential studies. Our procedure for evaluating records identified during the literature search followed the Preferred Reporting Items for Systematic Reviews and Meta-analyses (PRISMA) criteria [25]. Disagreements were resolved through discussion.

### Eligible criteria

We defined study eligibility using the PICO strategy (patient population, intervention, comparison, and outcomes) [26]. A study was considered relevant to this review according to the following criteria: 1) Adult patients with confirmed cancer, eligible for surgical treatment and undergoing SLN biopsy (SLNB) or regional lymphadenectomy; 2) patients did not undergo any neoadjuvant treatment; 3) the main objective was to compare OSNA using fresh LN with postoperative standard formalin-fixed paraffin-embedded (FFPE)-H&E analysis; 4) LN were dissected and analysed using both OSNA and the standard technique at the same time; 5) the pathological examination method was fully described; 6) results were reported per node (minimum 100 nodes); 7) sufficient data was available to calculate true-positive, false-positive, false-negative and true-negative values. We limited these criteria to English original studies. Review articles, meta-analysis, conference abstracts, and letters were excluded.

### Study selection

**Fig. 1:**
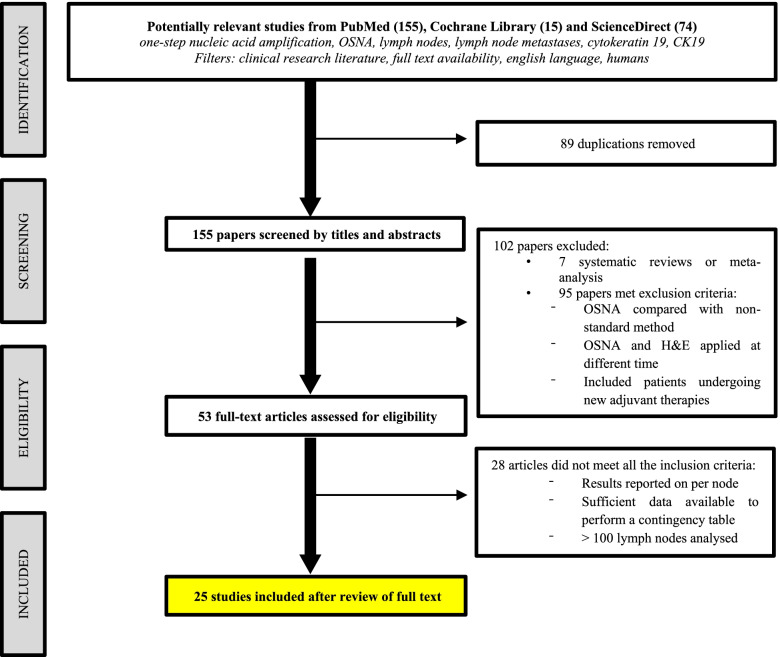
PRISMA flow diagram

The flow diagram of study selection process was outlined in Fig. 1. A total of 244 potentially relevant studies were identified using the searching terms described. Eighty-nine duplicated studies were initially excluded. After screening titles and abstracts, 102 papers were removed. From the remaining 52 studies, 28 were excluded after full text review because the comparison was made with intraoperative frozen section or touch imprint cytology as a reference method, less than 100 nodes were included, analysis was performed per patient, or insufficient data was available to form 2 × 2 tables.

Finally, 25 studies met all the requirements to be considered in the systematic review.

### Quality assessment

Quality Assessment of Diagnostic Accuracy Studies 2 (QUADAS-2) was used as an evidence-based quality assessment tool [27]. QUADAS-2 comprised four domains: patient selection, index test, reference standard, and flow and timing. The risk of bias of each study was evaluated by two independent reviewers as low “+”, high “-” or unclear “?” risk.

The QUADAS-2 results summarized in Table 2 suggest a low risk of bias and a moderate to high overall quality of all 25 included studies.

**Table 2:**
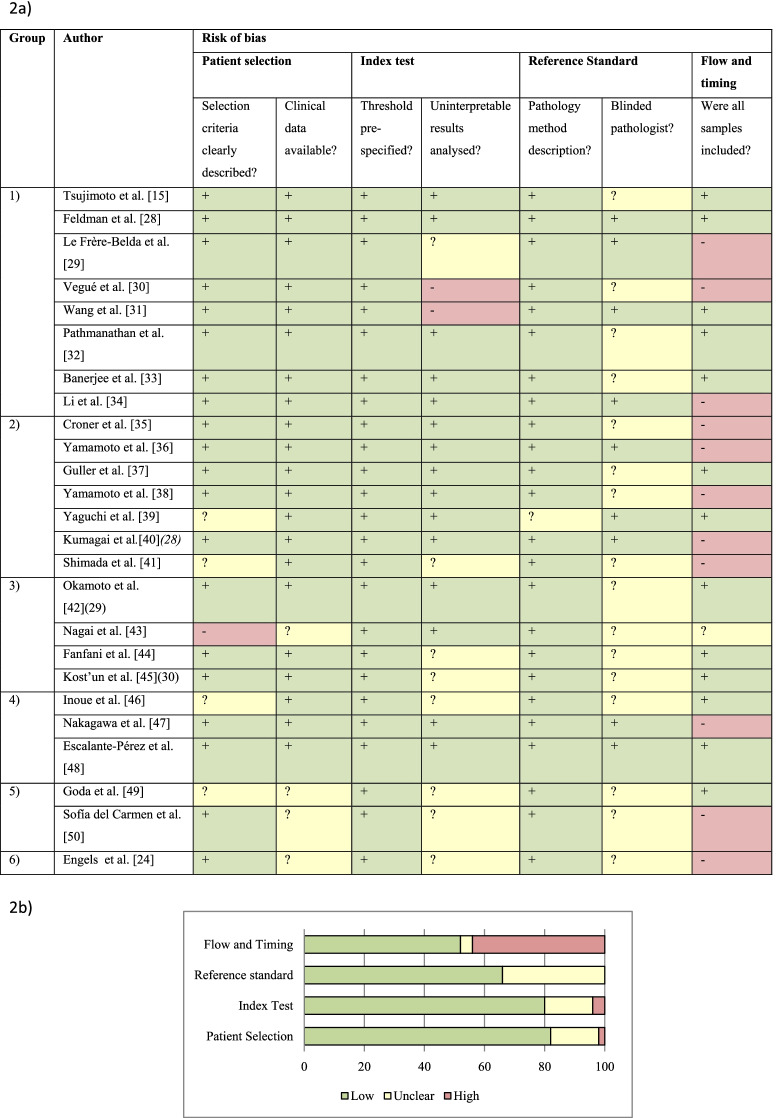
Risk of bias of included studies

a) Assessment of risk of bias. Summary of risk of bias for each study; +: low risk of bias; −: high risk of bias;?: unclear risk of bias

b) Risk of bias graph about each risk of bias item presented as percentages across all included studies

## Results

The 25 eligible studies have been published between January 2007 and March 2021. Our review included SLN and non-SLN from six different groups of tumours: 1) breast [15, 28–34], 2) gastrointestinal —colorectal [35–38] and gastric cancers [39–41]—, 3) gynecological —cervical [42] and endometrial cancers [43–45]—, 4) lung [46–48], 5) head and neck —head and neck squamous cell carcinomas (HNSCC) [49] and thyroid cancers [50]— and 6) PCa [24]. All studies were prospectively designed. OSNA was considered as index test and a threshold of 250 copies of CK19 mRNA per μL was fixed to differentiate between negative (< 250 copies/μL) and positive (≥250 copies/μL) results. OSNA was compared with post-operative FFPE tissue sections with H&E staining as the reference standard. Eleven studies also included also CK19 IHC analysis in addition to H&E staining and OSNA. A LN was cut into at least two parts (depending on LN size) and divided between OSNA assay and pathology. Contingency tables were created, and concordance rate was reported. Seventeen studies analysed the discordant cases (OSNA + / H&E -; OSNA - / H&E+) using different techniques.

Detailed characteristics are shown in Table 3.

**Table 3:** Characteristics of included studies

Group	Author	Year	Country	Tumour type	No. Patients	No. Nodes	Reference method	CK19 IHC	Analysis of discordant cases
1)	Tsujimoto et al. [15]	2007	Japan	Breast	101	325	H&E and IHC	yes	yes
	Feldman et al. [28]	2011	USA	Breast	496	1044	H&E and IHC	yes	yes
	Le Frère-Belda et al. [29]	2012	France	Breast	233	503	H&E and IHC	yes	yes
	Vegué et al. [30]	2012	Spain	Breast	57	567	H&E	yes	no
	Wang et al. [31]	2012	China	Breast	552	1188	H&E	no	yes
	Pathmanathan et al. [32]	2014	Australia	Breast	98	170	H&E and IHC	no	no
	Banerjee et al. [33]	2014	UK	Breast	170	268	H&E	no	no
	Li et al. [34]	2015	China	Breast	115	311	H&E	no	yes
2)	Croner et al. [35]	2010	Germany	Colorectal	184	184	H&E and IHC	yes	yes
	Yamamoto et al. [36]	2011	Japan	Colorectal	85	385	H&E	no	yes
	Guller et al. [37]	2012	Switzerland	Colorectal	22	313	H&E and IHC	yes	yes
	Yamamoto et al. [38]	2016	Japan	Colorectal	204	1925	H&E	no	no
	Yaguchi et al. [39]	2011	Japan	Gastric	32	162	H&E	no	yes
	Kumagai et al. [40]	2014	Japan	Gastric	61	394	H&E	no	yes
	Shimada et al. [41]	2019	Japan	Gastric	43	439	H&E	no	yes
3)	Okamoto et al. [42](29)	2013	Japan	Cervical	32	130	H&E	no	yes
	Nagai et al. [43]	2015	Japan	Endometrial	35	137	H&E	no	yes
	Fanfani et al. [44]	2018	Italy	Endometrial	40	110	H&E and IHC	yes	no
	Kost’un et al. [45](30)	2019	Czech Republic	Endometrial	58	135	H&E and IHC	yes	no
4)	Inoue et al. [46]	2012	Japan	Lung	49	165	H&E and IHC	no	no
	Nakagawa et al. [47]	2016	Japan	Lung	111	410	H&E	no	yes
	Escalante-Pérez et al. [48]	2019	Spain	Lung	160	705	H&E and IHC	yes	no
5)	Goda et al. [49]	2012	Japan	HNSCC	65	312	H&E	no	yes
	Sofía del Carmen et al. [50]	2016	Spain	Thyroid	37	267	H&E and IHC	yes	yes
6)	Engels et al. [24]	2021	Germany	Prostate	64	534	H&E and IHC	yes	yes

*CK19* Cytokeratin 19, *FN* False negative, *FP* False positive, *HNSCC* Head and neck squamous cell carcinoma, *H&E* Hematoxylin and eosin, *IHC* Immunohistochemistry, *No* Number of, *TN* True negative, *TP* True positive

Sensitivity, specificity, positive predictive value (PPV), negative predictive value (NPV), and concordance are listed in Table 4. Discordant cases are included in the reported results.

**Table 4:** OSNA accuracy compared with histopathological examination in included tumours

Group	Author	H&E positive		H&E negative		Sensitivity	Specificity	Concordance
		OSNA negative	OSNA positive	OSNA negative	OSNA positive			
1)	Tsujimoto et al. [15]	0.6 (2)	13.2 (43)	85 (276)	1.2 (4)	95.6	98.6	98.2 (319/325)
	Feldman et al. [28]	3 (31)	10.2 (107)	83.1 (868)	3.6 (38)	77.5	95.8	93.4 (975/1044)
	Le Frère-Belda et al. [29]	2.4 (12)	10.1 (51)	82.1 (413)	5.4 (27)	80.9	93.9	92.2 (464/503)
	Vegué et al. [30]	0 (0)	1.1 (6)	92.1 (522)	6.9 (39)	100	93	93.1 (528/567)
	Wang et al. [31]	2.6 (31)	13.4 (159)	78 (927)	6 (71)	83.7	92.9	91.4 (1086/1188)
	Pathmanathan et al. [32]	1.8 (3)	14.7 (25)	80.6 (137)	3 (5)	89.3	96.5	95.3 (162/170)
	Banerjee et al. [33]	0.7 (2)	14.6 (39)	81 (217)	3.7 (10)	95.1	95.6	95.5 (256/268)
	Li et al. [34]	2 (6)	9.6 (30)	85.5 (266)	2.9 (9)	83.3	96.7	95.2 (296/311)
2)	Croner et al. [35]	1.6 (3)	20.1 (37)	75.5 (139)	2.7 (5)	92.5	96.5	95.7 (176/184)
	Yamamoto et al. [36]	1 (4)	20.5 (79)	76.6 (295)	1.8 (7)	95.2	97.7	97.1 (374/385)
	Guller et al. [37]	0.6 (2)	16.3 (51)	79.6 (249)	3.5 (11)	96.2	95.7	95.8 (300/313)
	Yamamoto et al. [38]	1 (20)	6.5 (125)	89.2 (1717)	3.3 (63)	86.2	96.5	95.7 (1842/1925)
	Yaguchi et al. [39]	3.1 (5)	24.7 (40)	69.8 (113)	2.5 (4)	88.9	96.6	94.4 (153/162)
	Kumagai et al. [40]	2.3 (9)	11.4 (45)	82.7 (326)	3.6 (14)	83.3	95.9	94.2 (371/394)
	Shimada et al. [41]	1.8 (8)	3.2 (14)	93.9 (412)	1.1 (5)	63.6	98.8	97 (426/439)
3)	Okamoto et al. [42]	2.3 (3)	2.3 (3)	93.8 (122)	1.5 (2)	50	98.4	96.2 (125/130)
	Nagai et al. [43]	2.2(3)	10.2 (14)	86.9 (119)	0.7 (1)	82.4	99.2	97.1 (133/137)
	Fanfani et al. [44]	0.9 (1)	7.2 (8)	86.4 (95)	5.4 (6)	88.9	94.1	93.6 (103/110)
	Kost’un et al. [45]	0.7 (1)	7.4 (10)	78.5 (106)	13.3 (18)	90.9	85.5	85.9 (116/135)
4)	Inoue et al. [46]	0.6 (1)	11.5 (19)	87.3 (144)	0.6 (1)	95	99.3	98.8 (163/165)
	Nakagawa et al. [47]	2.9 (12)	11.5 (47)	81.2 (333)	4.4 (18)	79.7	94.5	92.7 (380/410)
	Escalante-Pérez et al. [48]	0.1 (1)	4.8 (34)	91.3 (644)	3.7 (26)	97.1	96.1	96.2 (678/705)
5)	Goda et al. [49]	2.6 (8)	17 (53)	77.2 (241)	3.2 (10)	86.9	96	94.2 (294/312)
	Sofía del Carmen et al. [50]	5.2 (14)	28.1 (75)	60 (160)	6.7 (18)	84.2	89.9	88 (235/267)
6)	Engels et al. [24]	2.2 (12)	14.2 (76)	80.5 (452)	3.4 (18)	91.4	100	98.8 (528/534)

Figures are expressed as percentages and (number of cases) in parentheses

*H&E* Hematoxylin and eosin, *No* Number of, *OSNA* One-step nucleic acid amplification

## Discussion

LN status is a key prognostic factor in the decision-making process of cancer management. For a long time, sectioning and H&E staining technique has been the gold standard for the evaluation of LN metastases. Even though it remains an adequate tool, some limitations have been described, especially low sensitivity in detecting the accurate tumour burden, mainly as a consequence of sampling bias [10], as well as a subjective and time-consuming result. To overcome these limitations, OSNA assay has been developed as a fast, objective, automated, and reproducible way to examine the whole LN, raising a general interest to explore its utility for lymphatic metastases identification in different tumours.

OSNA gives a quantitative result of CK19 mRNA copies, which is present in several simple epithelia but is not expressed in healthy lymphatic tissue [12]. CK19 was initially proposed as a marker for the detection of LN metastases in breast cancer, where it is found in up to 98% of cases [51]. In 2007, Tsujimoto et al. [15] determined 250 copies/μl as the optimum cut-off point to define a positive axillary LN in breast cancer population. Nonetheless, it is known that the number of positive LN and the size of metastases are significant prognostic factors in most tumours. Therefore, it was also established a second cut-off point of 5000 copies/μl to distinguish between micro and macrometastases [15]. Subsequent studies have confirmed these values and all the results reflected in this review are based on them.

In 2013 V. Peg et al. [21] defined the concept of total tumour load (TTL) as the total CK19 mRNA copies of all positive SLNs. TTL serves as a predictive and prognosis value, providing more accurate staging than pathological findings. Accordingly, different OSNA studies in breast SLN have set cut-off values in order to predict the axillary LN status; some of which (10,000–15,000 copies) are already included in clinical guidelines [52, 53]. In 2017, Rakislova et al. [54] explored its utility to predict recurrences in colorectal carcinoma, and a recent study confirmed that a TTL ≥ 6000 copies/μl was associated with worse disease free survival in those patients [55].

The analysis of SLN in breast cancer patients is still its main clinical application, but over the years OSNA has raised interest in the pathology community for a more accurate LN staging in other cancer entities. In the last decade, several reports comparing OSNA with histopathological examination have been published, but after a systematic review of the available literature, to date only two studies related to PCa have been found [23, 24].

All the articles included in this review compare OSNA assay with postoperative H&E staining in the same LN. There is a general concordance between OSNA and standard H&E of over 85%. No full information about discordant cases is available, but we have found not only different explanations for them but also heterogeneity in its analysis. Main justifications for the discordant cases are low or no tumour CK19 expression, tumour allocation bias (TAB) and contamination by other epithelial cells [46].

As CK19 is the single molecular marker used in OSNA assay, low tumour CK19 expression may result in a false-negative OSNA case. Different CK19 expression levels have been described for other malignancies such as colorectal (94.1%) [36], gastric (98.6%) [39], gynecological (98%) [43], lung (96%) [48], HNSCC (91.1%) [49] or PCa (100%) [23]. Moreover, certain tumour subtypes are more likely to the lack of CK19 expression, as observed in metaplastic and lobular breast carcinomas [56]. Interestingly, Goda et al. [49] performed a CK19 IHC in primary HNSCC as a first step when analyzing discordant results, detecting no expression of CK19 in 75% of those cases. Aiming to reduce false-negative cases in breast, thyroid and lung carcinomas, Vegué et al. [30], del Carmen et al. [50], and Escalante Pérez et al. [48] verified the presence of CK19 in primary tumour by IHC before LN analysis. For a more accurate interpretation of the results, we encourage future researchers to include CK19 expression in primary tumours as patient inclusion criteria.

When compared to histopathological examination, OSNA offers the advantage to obtain objective and quantitative data about tumour load of the whole LN in a fast and effortless way, avoiding interobserver variability. However, to properly compare both techniques on the same node, it is mandatory to split it, leading to a possible misdetection of metastasis by one of the methods, which is called TAB. Trying to justify the discrepancies by the need for sectioning, 17 studies have reported different strategies: second-round OSNA analysis, CK19 IHC, exhaustion of the paraffin-embedded SLN slices, CK19 Western blot, or CK19 qRT-PCR. Most of the metastases from the discrepancies were confirmed thanks to these strategies.

In contrast, a potential disadvantage of examining the whole LN with OSNA is that there is no tissue left for subsequent histopathological examination following complete homogenization. Nonetheless, RNA-based molecular tests are possible using OSNA lysate, thus allowing any follow-up molecular testing. Moreover, fresh lymphatic tissue requires a thorough dissection to avoid missing nodes, which must be completely separated from fat tissue by a trained pathologist. It is also important to note that in the case of a coexisting neoplasm, OSNA cannot define which primary tumour the metastases come from and is unable to distinguish LN tumour cells from other benign epithelial inclusions. Therefore, it is imperative to exclude other cancers with the same lymphatic drainage and avoid contaminations.

Despite these concerns, the results reported show a high specificity, concordance rate, and NPV of OSNA assay when compared with the H&E method. Specifically, in breast cancer patients, that high NPV provides enough evidence to become the gold standard for SLN evaluation. As slicing is required for the analysis per node, a concordance of 100% cannot be achieved due to Tab. A high concordance rate of over 85% suggests that OSNA could be an alternative technique to histopathological examination in terms of its ability to detect LN metastases.

Regarding PCa, CK19 has been found not only in neoplastic tissue but also in basal and luminal cells of normal, dysplastic and benign hyperplastic tissues, although complete data concerning CK19-RNA levels is still missing [57]. In 2018, Winter et al. [23] published the first OSNA assay in PCa. A total of 20 primary PCa tumours from intermediate-high risk PCa patients (Gleason ≥7) were analysed. A central slice was analysed by OSNA, while surrounding slices were sent for both conventional H&E staining and CK19 IHC. PCa was confirmed by H&E in all 20 samples; OSNA was able to detect CK19 mRNA in 100% of cases, ranging from 320 to 250.000copies/μl while IHC did not detect CK19 in one specimen. Given the small sample size and the high tumour burden of the selected patients, we cannot fully extrapolate these findings for all PCa patients. Recently, Engels et al. [24] published an assay that verifies the reliability of OSNA for the first time in PCa. A total of 574 SLNs from 64 PCa patients undergoing prostatectomy and sentinel lymphadenectomy were included. SLNs were assessed by conventional H&E staining and OSNA assay. The comparison between both techniques showed a sensitivity, specificity and concordance rates of 84.2, 96.1 and 94.4%, respectively, concluding that OSNA assay provides an accurate diagnosis that might improve LN staging in PCa.

To date, the ePLND remains the most accurate staging procedure [58], but individual assessment of all dissected LNs is laborious and time consuming. Using OSNA, LNs can be pooled together and analysed in a few samples as already done in colorectal cancer [54]. In fact, Engels et al. [24] suggested that such approach could be feasible to analyse PCa LNs as a faster and economic alternative.

Based on available evidence, current European PCa Guidelines [58] state a weak recommendation of offering adjuvant therapy to pN1 PCa patients with ≥2 positive LN after radical prostatectomy with ePLND, especially in cases with higher pathologic grade, as they have an increased risk of biochemical recurrence. To that point, post-operative OSNA analysis and the possibility to define a TTL value might be helpful in the identification of this subgroup of patients suitable for adjuvant therapy. Moreover, intraoperative use of OSNA may be a great opportunity to set up sentinel-guided LN dissection in PCa. Future investigations may bring light to the clinical impact of OSNA in PCa as well as to its potential predictive and prognostic roles.

## Conclusion

OSNA is a suitable tool to standardize LN evaluation in most CK19 expressing tumours due to the possibility to analyse the whole LN in a fast, objective, automated, and reproducible way. According to limited data available, OSNA assay has also demonstrated a high diagnostic accuracy for the detection of LN tumour burden in PCa, but more studies are needed to confirm its validation.

## Data Availability

All the data and materials analysed are included in the main paper. Further data are available from the corresponding author on reasonable request.

## Abbreviations

CK19: Cytokeratin 19
ePLND: Extended pelvic lymph node dissection
FFPE: Formalin-fixed paraffin-embedded
HNSCC: Head and neck squamous cell carcinomas
H&E: Haematoxylin and eosin
IHC: Immunohistochemistry
LN: Lymph node
OSNA: One-step nucleic acid amplification
PCa: Prostate cancer
PSA: Prostate specific antigen
PRISMA: Preferred reporting items for systematic reviews and meta-analyses
QUADAS 2: Quality assessment of diagnostic accuracy studies 2
RT-PCR: Reverse transcription-polymerase chain reaction
SLN: Sentinel lymph node
TAB: Tumour allocation bias
TTL: Total tumour load
